# Supplementary figures and images for: Kinesin-8B controls basal body function and flagellum formation and is key to malaria transmission
Source: Life Sci Alliance. 2019 Aug 13;2(4):e201900488. doi: 10.26508/lsa.201900488 (PMC6696982; doi:10.26508/lsa.201900488)

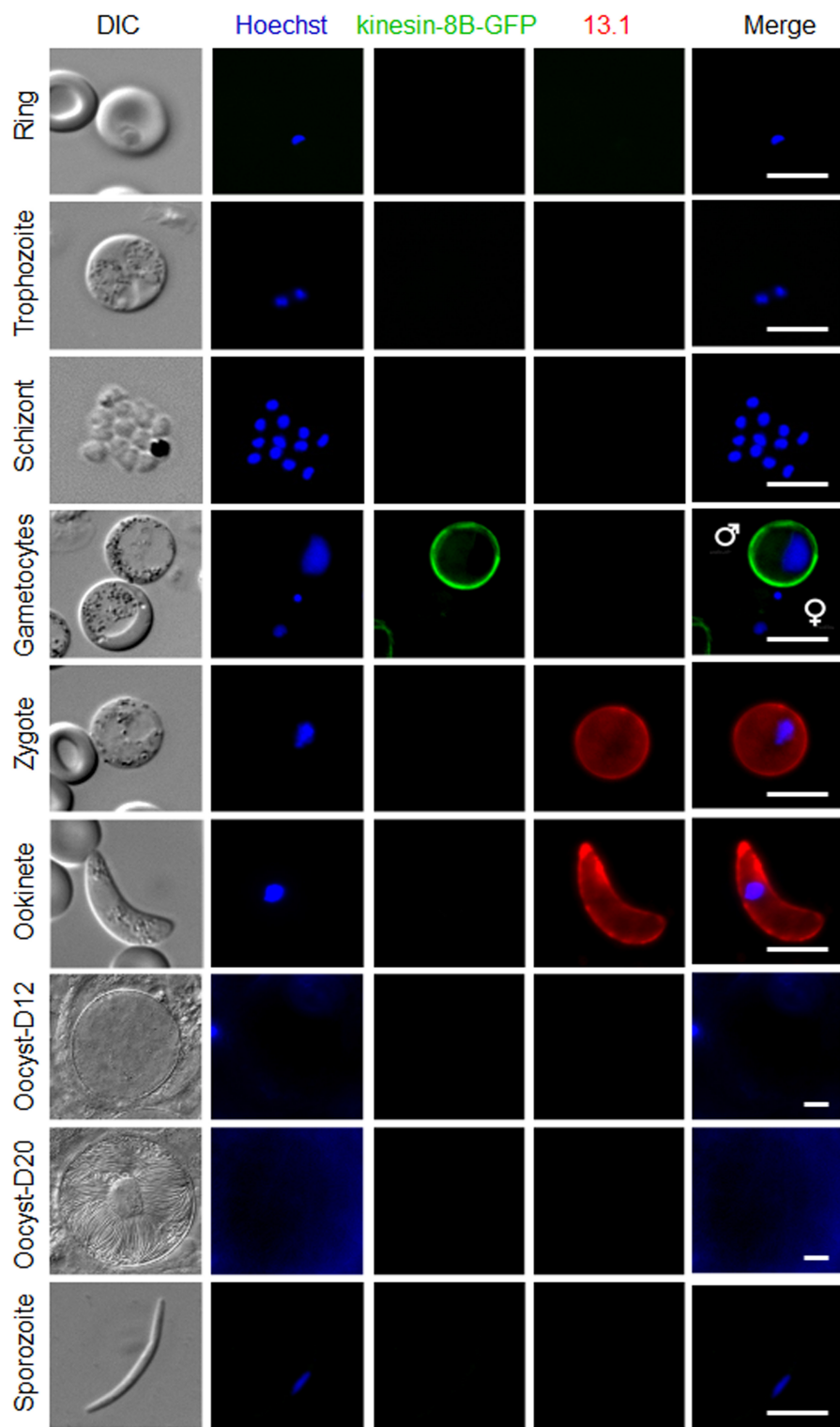

Supplement: Supplementary file 3 [file LSA-2019-00488_SdataFS3_1.pdf]
