# Supplementary material for: Kinesin-8B controls basal body function and flagellum formation and is key to malaria transmission
Source: Life Sci Alliance. 2019 Aug 13;2(4):e201900488. doi: 10.26508/lsa.201900488 (PMC6696982; doi:10.26508/lsa.201900488)
Supplement: Supplementary file 4 [file LSA-2019-00488_SdataFS3_2.pptx]

## Slide 1
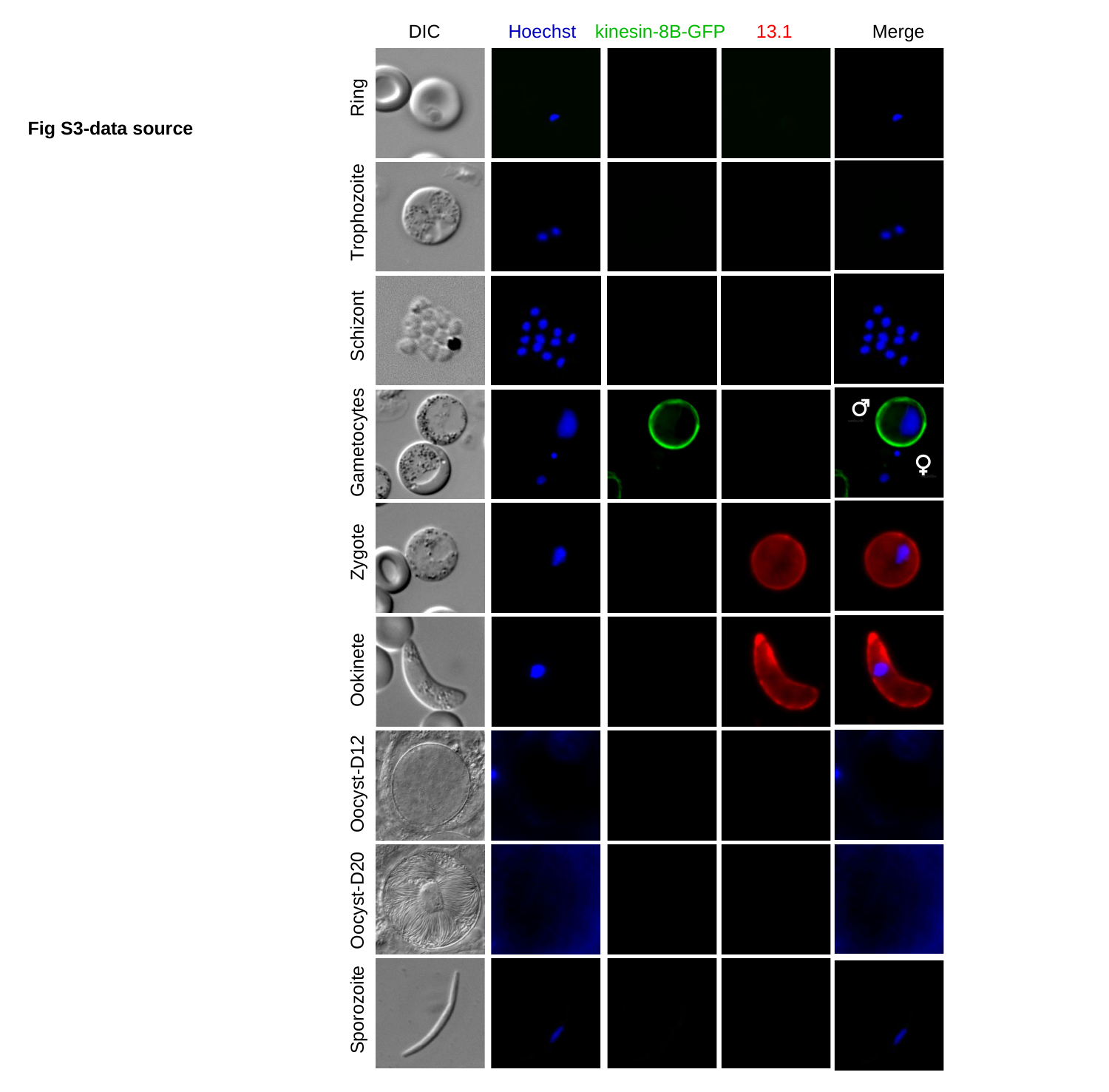

DIC
Hoechst
kinesin-8B-GFP
13.1
Merge
Ring
Trophozoite
Schizont
Gametocytes
Zygote
Ookinete
Oocyst-D12
Oocyst-D20
Sporozoite
Fig S3-data source
